# Comparative Genomics Provides Insight into the Function of Broad-Host Range Sponge Symbionts

**DOI:** 10.1128/mBio.01577-21

**Published:** 2021-09-14

**Authors:** Samantha C. Waterworth, Shirley Parker-Nance, Jason C. Kwan, Rosemary A. Dorrington

**Affiliations:** a Division of Pharmaceutical Sciences, University of Wisconsin, Madison, Wisconsin, USA; b Department of Biochemistry and Microbiology, Rhodes University, Makhanda, South Africa; c South African Environmental Observation Network, Elwandle Coastal Node, Gqeberha (Port Elizabeth), South Africa; d South African Institute for Aquatic Biodiversity, Makhanda, South Africa; CEH-Oxford

**Keywords:** *Latrunculiidae*, *Tethybacterales*, *Poribacteria*, symbiosis, *Porifera*, comparative genomics

## Abstract

The fossil record indicates that the earliest evidence of extant marine sponges (phylum Porifera) existed during the Cambrian explosion and that their symbiosis with microbes may have begun in their extinct ancestors during the Precambrian period. Many symbionts have adapted to their sponge host, where they perform specific, specialized functions. There are also widely distributed bacterial taxa such as *Poribacteria*, *SAUL*, and *Tethybacterales* that are found in a broad range of invertebrate hosts. Here, we added 11 new genomes to the *Tethybacterales* order, identified a novel family, and show that functional potential differs between the three *Tethybacterales* families. We compare the *Tethybacterales* with the well-characterized *Entoporibacteria* and show that these symbionts appear to preferentially associate with low-microbial abundance (LMA) and high-microbial abundance (HMA) sponges, respectively. Within these sponges, we show that these symbionts likely perform distinct functions and may have undergone multiple association events, rather than a single association event followed by coevolution.

## INTRODUCTION

While their exact time of origin is a subject of hot debate, fossil records indicate that extant marine sponges (phylum *Porifera*) existed during the Cambrian explosion, approximately 540 million years ago (1, 2), and that symbiotic relationships with microbes may have begun even before the evolution of extant sponge taxa (3). This would suggest that sponges may be one of the oldest living examples of symbiotic relationships. Sponges are remarkably efficient filter feeders, acquiring nutrients via phagocytosis of particulate matter, compromising mainly microbes, from the surrounding water (4). Since their emergence approximately 540 million years ago, sponges have evolved close associations with microbial symbionts that provide services essential for the fitness and survival of the host in diverse ecological niches (1, 5, 6). These symbionts are involved in a diverse array of beneficial processes, including the cycling of nutrients (7, 8) such as nitrogen (8–13), sulfur (14, 15), and phosphate (16, 17), the acquisition of carbon (18, 19), and a supply of vitamins (20–23) and amino acids (20). They can play a role in the host sponge life cycle, such as promoting larval settlement (24). In addition, some symbionts provide chemical defenses against predators and biofouling through the production of bioactive compounds (25–28). In turn, the sponge host can provide symbionts with nutrients and minerals, such as creatinine and ammonia as observed in Cymbastela concentrica sponges (11).

As filter-feeders, sponges encounter large quantities of bacteria and other microbes. How sponges are able to distinguish between prey bacteria and those of potential benefit to the sponge, and the establishment of symbiotic relationships, is still not well understood, but the structure and composition of bacterial lipopolysaccharide, peptidoglycan, or flagellin may aid the host sponge in distinguishing symbionts from prey (29). Sponge hosts encode an abundance of nucleotide-binding domain and leucine-rich repeat (NLR) receptors, which recognize different microbial ligands and potentially allow for distinction between symbionts, pathogens, and prey (30). Additionally, it has recently been shown that phages produce ankyrins which modulate the sponge immune response and allow for colonization by bacteria (31).

Symbionts are often specific to their sponge host with enriched populations relative to the surrounding seawater (32). However, there are a small number of “cosmopolitan” symbionts that are ubiquitously distributed across phylogenetically distant sponge hosts. The *Poribacteria* and “*sponge-associated unclassified lineage*” (*SAUL*) (33) are examples of such cosmopolitan bacterial species. Phylum *Poribacteria* bacteria were thought to be exclusively found in sponges (34). However, the identification of 13 putative *Poribacteria*-related metagenome-assembled genomes (MAGs) from ocean water samples (35) led to the reclassification and distinction of sponge-associated *Entoporibacteria* and free-living *Pelagiporibacteria* within the phylum (36). *Entoporibacteria* are associated with phylogenetically divergent sponge hosts in distant geographic locations, with no apparent correlations between their phylogeny and that of their sponge host or location (36, 37). Different *Poribacteria* phylotypes have been detected within the same sponge species (38). The *Entoporibacteria* carry several genes (39, 40) that encode enzymes responsible for the degradation of carbohydrates and metabolism of sulfates and uronic acid (41–43), prompting the hypothesis that these bacteria may be involved in the breakdown of the proteoglycan host matrix (43). However, subsequent analyses of *Poribacteria* transcriptomes from the mesohyl of Aplysina aerophoba sponges showed that genes involved in carbohydrate metabolism were not highly expressed (42). Instead, there was a higher expression of genes involved in 1,2-propanediol degradation and import of vitamin B_12_, which together suggest that the bacterium may import vitamin B_12_ as a necessary cofactor for anaerobic 1,2-propanediol degradation and energy generation (42).

The *SAUL* bacteria belong to the larger taxon of candidate phylum *PAUC34f* and have been detected, although at low abundance, in several sponge species (44). Host-associated *SAUL* bacteria were likely acquired by eukaryotic hosts (sponges, corals, tunicates) at different evolutionary time points and are phylogenetically distinct from their planktonic relatives (44). Previous investigations into the only two *SAUL* bacterial genomes provided evidence to suggest that these symbionts may play a role in the degradation of host and algal carbohydrates, as well as the storage of phosphate for the host during periods of phosphate limitation (33).

Recently, a third group of ubiquitous sponge-associated betaproteobacterial symbionts were described (45). The proposed new order, the *Tethybacterales*, comprises two families, the *Tethybacteraceae* and the *Persebacteraceae* (45). Based on assessment of MAGs representative of different species within these two families, it was shown that the bacteria within these families had functionally radiated, as they coevolve with their specific sponge host (45). The *Tethybacterales* are distributed both globally and with phylogenetically diverse sponges that represent both high-microbial abundance (HMA) and low-microbial abundance (LMA) sponges (45). These bacteria have also been detected in other marine invertebrates, ocean water samples, and marine sediment (45), suggesting that these symbionts may have, at one point, been acquired from the surrounding environment.

*Tethybacterales* are conserved in several sponge microbiomes (46) and can be the numerically dominant bacterial population (15, 47–54), with some predicted to be endosymbiotic (15, 47, 52). In Amphimedon queenslandica, the AqS2 symbiont, Amphirhobacter heronislandensis (family *Tethybacteraceae*), is codominant with sulfur-oxidizing *Gammaproteobacteria* AqS1. *A. heronislandensis* AqS2, is present in all stages of the sponge life cycle (55) and appears to have a reduced genome (15). Interestingly, the AqS2 MAG shares some functional similarity with the codominant AqS1, including the potential to generate energy via carbon monoxide oxidation, assimilate sulfur, and produce most essential amino acids (15). However, these sympatric symbionts differ significantly in what metabolites they could possibly transport (15).

In this study, we used the dominant, conserved *Tethybacterales* (strain Sp02-1) symbiont of *Tsitsikamma* (subgenus *Tsitsikamma*) *favus* sponge species (family *Latrunculiidae*) (56–60) as a springboard into a deeper investigation of *Tethybacterales*. Here, we report a comparative study using new and existing *Tethybacterales* genomes and show that functional potential follows that of their taxonomic ranking rather than host-specific adaptation. We also show that the *Tethybacterales* and *Poribacteria* have distinct functional repertoires, that these bacterial families can coexist in a single host, and that the *Tethybacterales* may represent a more ancient lineage of ubiquitous sponge-associated symbionts.

## RESULTS AND DISCUSSION

The microbiomes of sponges of the *Latrunculiidae* family are highly conserved and are dominated by populations of related betaproteobacterial symbionts. These bacteria have since been reclassified as class *Gammaproteobacteria*, as several betaproteobacteria were, when genome phylogeny was proposed as the basis for taxonomy, which has since been incorporated in the Genome Taxonomy Database (GTDB) (61). The numerically dominant symbiont in *T.* (*T.*) *favus* sponges is strain Sp02-1. Based on their 16S rRNA gene sequence, the Sp02-1 strain and closely related symbionts from different latrunculid sponges are likely members of the newly described *Tethybacterales* order.

### Characterization of the putative *Tethybacterales* genome bin.

Genome bin 003B_4, from sponge specimen TIC2018-003B, included a 16S rRNA gene sequence that shared 99.86% identity with the *T.* (*T.*) *favus*-associated *Tethybacterales* Sp02-1 (previously called the betaproteobacterium Sp02-1 [56, 57]) full-length 16S rRNA gene clone (GenBank accession number HQ241787.1). The next-closest relatives were uncultured 16S clones from Xestospongia muta and Tethya aurantium sponges (Fig. S2). Bins 050A_14, 050C_6, and 003D_6 were also identified as possible representatives of *Tethybacterales* Sp02-1 based on their predicted phylogenetic relatedness. However, they were of low quality and were not used in downstream analyses.

10.1128/mBio.01577-21.2 FIG S2 Phylogeny of the *T.* (*T.*) *favus*-associated *Tethybacterales* bin. The phylogenetic relationship between the putative Sp02-1 genome and closest relatives was based on 16S rRNA sequences and inferred using the UPGMA method. The percentages of replicate trees in which the associated taxa clustered together in the bootstrap test (10,000 replicates) are shown next to the branches. The tree is drawn to scale, with branch lengths in the same units as those of the evolutionary distances used to infer the phylogenetic tree. The evolutionary distances were computed using the maximum composite likelihood method and are in the units of the number of base substitutions per site. This analysis involved 17 16S rRNA gene sequences with a total of 1,291 positions analyzed. All ambiguous positions were removed for each sequence pair (pairwise deletion option). Evolutionary analyses were conducted in MEGA X. Download FIG S2, EPS file, 0.6 MB. Copyright © 2021 Waterworth et al. 2021 Waterworth et al. https://creativecommons.org/licenses/by/4.0/ This content is distributed under the terms of the Creative Commons Attribution 4.0 International license.

Genome bin 003B_4 was used as a representative of the *Tethybacterales* Sp02-1 symbiont. Bin 003B_4 is approximately 2.95 Mbp in size and of medium quality per MIMAG standards (62) (Table S1), and it has a notable abundance of pseudogenes (∼25% of all genes), which resulted in a coding density of 65.27%, far lower than the average for bacteria (63). An abundance of pseudogenes and low coding density are usually indications that the genome in question may be undergoing genome reduction (64), similar to other genomes in the proposed order of *Tethybacterales* (45).

10.1128/mBio.01577-21.4 TABLE S1 Metadata and taxonomic classification of all bins/genomes used in this study. Download Table S1, TXT file, 0.1 MB. Copyright © 2021 Waterworth et al. 2021 Waterworth et al. https://creativecommons.org/licenses/by/4.0/ This content is distributed under the terms of the Creative Commons Attribution 4.0 International license.

The *Tethybacterales* Sp02-1 genome carries all genes necessary for glycolysis and PRPP biosynthesis, and most genes required for the citrate cycle and oxidative phosphorylation were detected in the gene annotations. Also present are the genes necessary to biosynthesize valine, leucine, isoleucine, tryptophan, phenylalanine, tyrosine, and ornithine amino acids, as well as genes required for transport of l-amino acids, proline, and branched amino acids. This would suggest that this bacterium may exchange amino acids with the host, as observed previously in both insect and sponge-associated symbioses (11, 65, 66).

A total of 13 genes unique to the *Tethybacterales* Sp02-1 symbiont were identified (i.e., not identified elsewhere in the *T. [T.] favus* metagenomes). One gene was predicted to encode an ABC transporter permease subunit that was likely involved in glycine betaine and proline betaine uptake. A second gene encoded 5-oxoprolinase subunit PxpA (Table S5). The presence of these two genes suggests that the *Tethybacterales* Sp02-1 genome can acquire proline and convert it to glutamate (67) in addition to glutamate already produced via glutamate synthase. Other unique genes encode a restriction endonuclease subunit and site-specific DNA-methyltransferase, which would presumably aid in defense against foreign DNA. At least seven of the unique gene products are predicted to be associated with phages, including the antirestriction protein ArdA. ArdA is a protein that has previously been shown to mimic the structures of DNA normally bound by type I restriction modification enzymes, which prevent DNA cleavage, and effectively results in antirestriction activity (68). If functionally active in the *Tethybacterales* Sp02-1 symbiont, we speculate that this protein may similarly prevent DNA cleavage through its mimicry of the targeted DNA structures and protect the genome against type I restriction modification enzymes. Finally, two of the unique genes were predicted to encode an ankyrin repeat domain-containing protein and a von Willebrand factor type A (VWA) domain-containing protein. These two proteins are known to be involved in cell-adhesion and protein-protein interactions (69, 70), and if active within the symbiont, they may help facilitate the symbiosis between the *Tethybacterales* Sp02-1 symbiont and the sponge host.

10.1128/mBio.01577-21.8 TABLE S5 Genes unique to the putative *Betaproteobacteria* symbiont Bin 003B4, relative to all other genes present in the four *T. favus* metagenomes. Download Table S5, TXT file, 0.002 MB. Copyright © 2021 Waterworth et al. 2021 Waterworth et al. https://creativecommons.org/licenses/by/4.0/ This content is distributed under the terms of the Creative Commons Attribution 4.0 International license.

### Comparison of putative Sp02-1 with other *Tethybacterales*.

Several *Tethybacterales* sponge symbionts have been described to date, and these bacteria are thought to have functionally diversified following the initiation of their ancient partnership (45). To test this hypothesis, we downloaded 12 genomes/MAGs of *Tethybacterales* (classified as AqS2 in GTDB) from the JGI database. Additionally, we assembled and binned metagenomic data from 36 sponge SRA data sets, covering 14 sponge species, and recovered an additional 14 AqS2-like genomes. Of the total 27 bins, 10 were of low quality, so Bin 003B_4 (Sp02-1) and 16 medium-quality *Tethybacterales* bins/genomes were used for further analysis (Table 1).

**TABLE 1:** General characteristics of putative *Tethybacterales* genomes/MAGs

Genome	Sponge host	Size (Mbp)	Complete (%)	Contam (%)	Quality	“Core” genes (%)	Study or reference Accession no.
“*Candidatus* Ukwabelana africanus” (003B_4)	*Tsitsikamma* (*T.*) *favus*	2.96	72.92	3.56	Medium	84.52	This study
“*Candidatus* Regalo mexicanus” (ImetM1_9)	Iophon methanophila	1.56	85.58	0.61	Medium	83.33	This study
“*Candidatus* Regalo mexicanus” (ImetM2_1_1)	*Iophon methanophila*	1.60	84.36	0.61	Medium	86.9	This study
Persebacter sydneyensis (C29)	Crella incrustans	1.52	82.87	0.61	Medium	78.57	45
							JGI 2784132075
Tethybacter castellensis (ccb2r)	Cymbastela concentrica	1.69	81	0.3	Medium	85.71	45
							JGI 2784132054
Beroebacter blanensis (Crambe1)	Crambe crambe	2.25	80.38	1.81	Medium	73.8	45
							JGI 2814122900
Amphirhobacter heronislandensis (AqS2)	Amphimedon queenslandica	1.61	71.13	1.52	Medium	80.95	15
							NCBI GCA_001750625.1
Calypsobacter congwongensis (B3)	*Scopalina* sp.	1.05	64.33	0.04	Medium	53.57	45
							JGI 2784132034
Telestobacter tawharauni (TSB1)	Tethya stolonifera	1.24	56.3	0	Medium	57.14	45
							JGI 2784132053
“*Candidatus* Hadiah malacca” (Csing_1)	Coelocarteria singaporensis	1.58	65.47	0.61	Medium	73.81	JGI 3300007741_3
“*Candidatus* Hadiah malacca” (Csing_2)	*Coelocarteria singaporensis*	1.36	58.12	0	Medium	72.62	JGI 3300007056_3
“*Candidatus* Hadiah malacca” (Csing_3)	*Coelocarteria singaporensis*	1.45	57.04	0.61	Medium	76.19	JGI 3300007046_3
“*Candidatus* Hadiah malacca” (Csing_5)	*Coelocarteria singaporensis*	1.16	55.82	0.61	Medium	75.00	JGI 3300021544_3
“*Candidatus* Hadiah malacca” (Csing_6)	*Coelocarteria singaporensis*	1.37	58.12	0	Medium	72.62	JGI 3300021545_3
“*Candidatus* Hadiah malacca” (Csing_7)	*Coelocarteria singaporensis*	0.98	54.76	1.93	Medium	69.05	JGI 3300021549_5
“*Candidatus* Donum taiwanensis” (CCyA_2_3)	*Cinachyrella* sp.	3.08	84.92	1.83	Medium	78.57	This study
“*Candidatus* Donum taiwanensis” (CCyB_3_2)	*Cinachyrella* sp.	3.45	79.58	5.93	Medium	75.00	This study
CCyA_16_0	*Cinachyrella* sp.	0.86	16.95	3.81	Low	35.71	This study
CCyA_3_0	*Cinachyrella* sp.	0.62	29.31	0	Low	15.48	This study
CCyA_3_39	*Cinachyrella* sp.	0.41	22.01	4.88	Low	36.90	This study
CCyB_501_11	*Cinachyrella* sp.	0.04	7.51	0	Low	17.86	This study
CCyB_502_83	*Cinachyrella* sp.	0.43	17.01	0	Low	21.43	This study
CCyC_2_7	*Cinachyrella* sp.	1.81	47.5	0	Low	57.14	This study
050C_6	*Tsitsikamma favus*	2.21	24.03	4.51	Low	32.14	This study
Csing_4	*Coelocarteria singaporensis*	1.08	39.35	0.61	Low	54.76	JGI 3300007053_5
050A_14	*Tsitsikamma* (*T.*) *favus*	6.08	61	19.4	Low	73.81	This study
003D_6	*Tsitsikamma* (*T.*) *favus*	0.33	0	0	Low	0	This study

**FIG 1:**
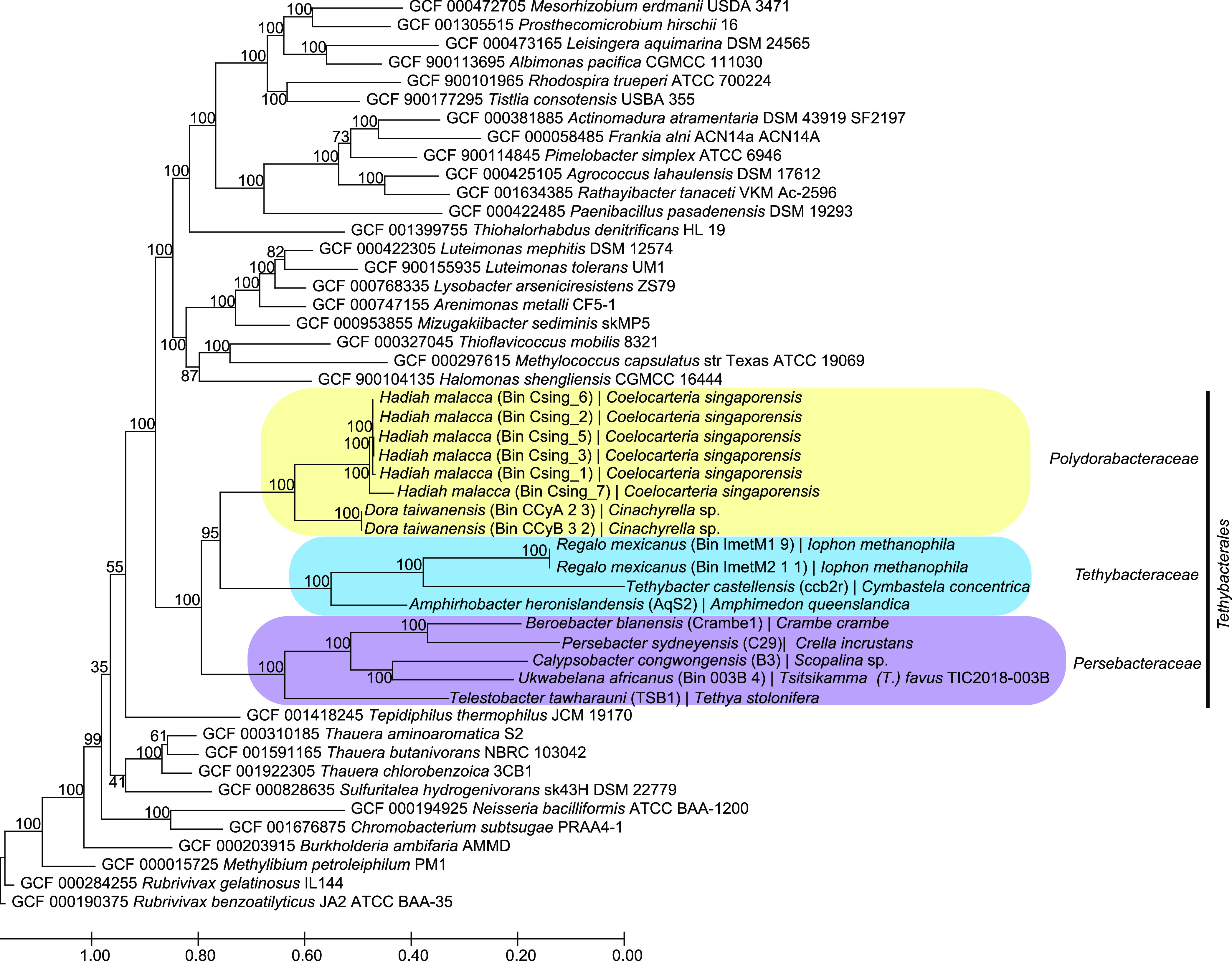
Phylogeny of the *Tethybacterales* sponge symbionts. Using autoMLST, single-copy markers were selected and used to delineate the phylogeny of these sponge-associated betaproteobacteria, revealing a new family of symbionts in the *Tethybacterales* order. Additionally, it was shown that the *T.* (*T.*) *favus*-associated Sp02-1 symbiont belongs to the *Persebacteraceae* family. The phylogenetic tree was inferred using the *de novo* method in AutoMLST using a concatenated alignment with IQ Tree and ModelFinder enabled. Branch lengths are proportional to the number of substitutions per site.

First, the phylogeny of the *Tethybacterales* symbionts was determined using single-copy marker genes in autoMLST, revealing a deep branching clade of these sponge-associated symbionts and revealing that bin 003B_4 clustered within the proposed *Persebacteraceae* family (Fig. 1). All members of the *Persebacteraceae* family dominate the microbial community of their respective sponge hosts (47, 56, 57, 71). We additionally identified what appears to be a third family, consisting of symbionts associated with Coelocarteria singaporensis and *Cinachyrella* sponge species (Fig. 1). Assessment of shared average amino acid identity (AAI) indicates that these genomes represent a new family, sharing an average of 80% AAI within the family (Table S6) (72). These three families share less than 89% sequence similarity with respect to their 16S rRNA sequences, with intraclade differences of less than 92% (Table S6). Therefore, they may represent novel classes (72) within the *Tethybacterales* order. While it is still hotly debated whether MAGs should be named at the genus level (73–76), we chose to tentatively name the additional genera and family after Oceanids of Greek mythology in keeping with Taylor and colleagues, who initially resolved the *Tethybacterales* order (45). We propose the family name *Polydorabacteraceae*, which means “many gifts.” Additionally, we propose species names for the newly identified genera as follows: Bin 003B_4 is a single representative of “*Candidatus* Ukwabelana africanus,” Bin Imet_M1_9 and Bin ImetM2_1_1 are both representatives of “*Candidatus* Regalo mexicanus,” Bin CCyA_2_3 and CCyB_3_2 are both representatives of “*Candidatus* Dora taiwanensis,” and all six bins from *C. singaporensis* are representative of “*Candidatus* Hadiah malacca.” In each case, the genus name means “gift from” in the local language (where possible) from where the host sponge was collected, and the species name reflects the region/country from which the sponge host was collected.

10.1128/mBio.01577-21.9 TABLE S6 Pairwise average amino acid identity scores (top) and pairwise average 16S rRNA gene sequence identity scores (bottom) between *Tethybacterales* genomes. Download Table S6, TXT file, 0.005 MB. Copyright © 2021 Waterworth et al. 2021 Waterworth et al. https://creativecommons.org/licenses/by/4.0/ This content is distributed under the terms of the Creative Commons Attribution 4.0 International license.

**FIG 2:**
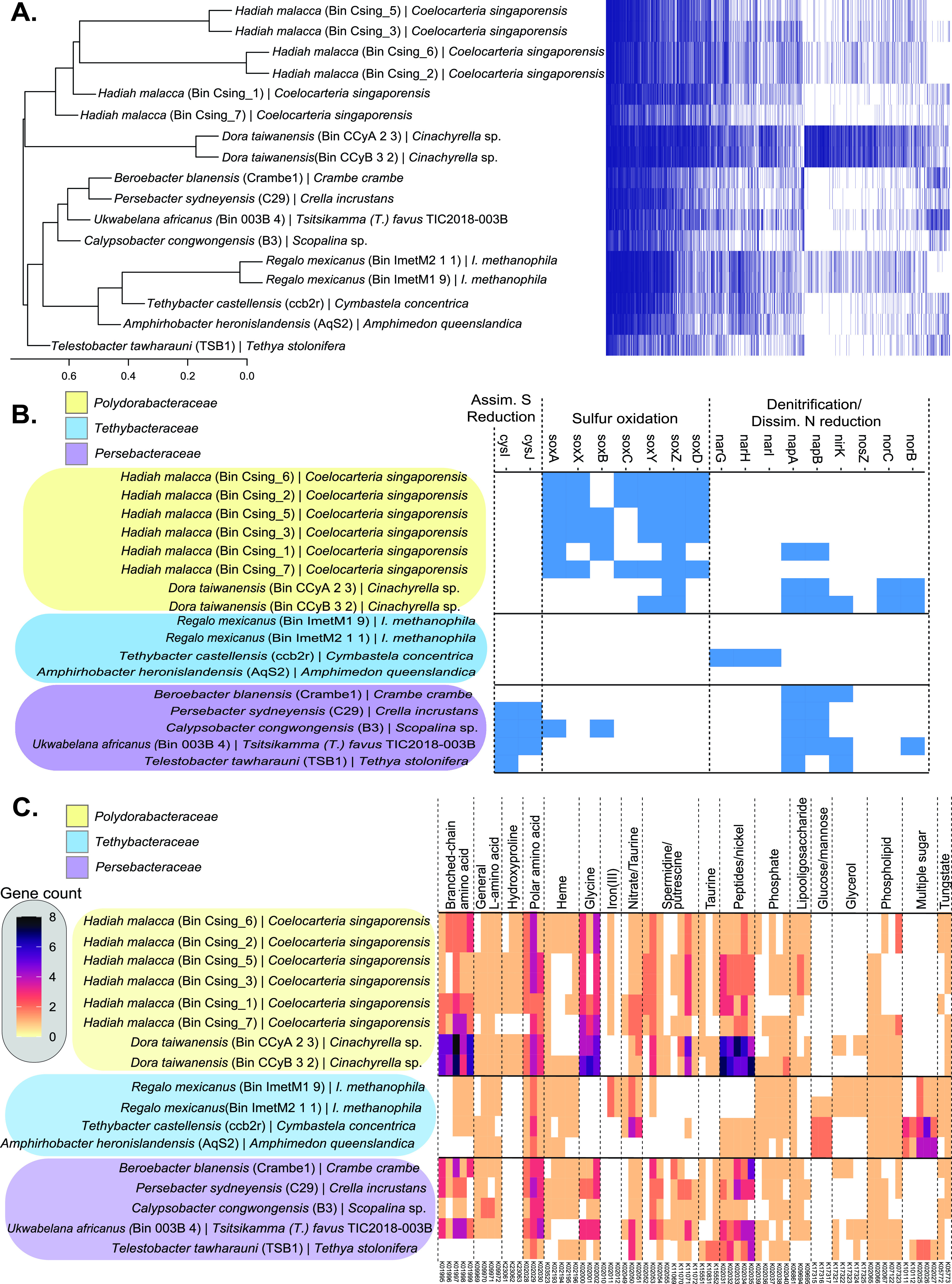
Functional specialization of *Tethybacterales* families. The newly proposed *Tethybacterales* order appears to consist of three bacterial families. (A to C) These families appear to have similar gene distribution (A), where the potential function of these genes indicates specialization in nutrient cycling (B) and solute transport (C).

We identified 4,306 groups of orthologous genes between all 17 *Tethybacterales* genomes, with only 18 genes common to all the genomes. More shared genes were expected, but as several of the genomes investigated are incomplete, it is possible that additional common genes would be found if the genomes were complete. Hierarchical clustering of gene presence/absence data revealed that the gene pattern of Bin 003B_4 most closely resembled that of *Tethybacterales* genomes from Crambe crambe, Crella incrustans, and the *Scopalina* sp. sponges (family *Persebacteraceae*) (Fig. 2A). A total of 13 of the shared genes between all *Tethybacterales* genomes encoded ribosomal proteins or those involved in energy production. Genes encoding chorismate synthase were found across all 17 genomes and suggest that tryptophan production may be shared among these bacteria. According to a recent study, Dysidea etheria and *A. queenslandica* sponges cannot produce tryptophan (a possible essential amino acid), which may indicate a common role for the *Tethybacterales* symbionts as tryptophan producers (77). Several other shared genes were predicted to encode proteins involved in stress responses, including protein-methionine-sulfoxide reductase, ATP-dependent Clp protease, and chaperonin enzyme proteins, which aid in protein folding or degradation under various stressors (78–82). Internal changes in oxygen levels (83) and temperature changes (84–86) are examples of stressors experienced by the sponge holobiont. It is unsurprising that this clade of largely sponge-specific *Tethybacterales* share the ability to deal with these many stressors as they adapt to their fluctuating environment.

Alignment against the KEGG database revealed some noteworthy trends that differentiated the three *Tethybacterales* families (Fig. 2B; Table S7): (i) the genomes of the proposed *Polydorabacteraceae* family include several genes associated with sulfur oxidation; (ii) the *Persebacteraceae* are unique in their potential for reduction of sulfite (*cysIJ*), and (iii) the *Tethybacteraceae* have the potential for cytoplasmic nitrate reduction (*narGHI*), while the other two families may perform denitrification. Similarly, the families differ to some extent in what can be transported in and out of the symbiont cell (Fig. 2C). Proposed members of the *Polydorabacteraceae* appear exclusively capable of transporting hydroxyproline, which may imply a role in collagen degradation (87). The *Tethybacteraceae* and *Persebacteraceae* appear able to transport spermidine, putrescine, taurine, and glycine, which in combination with their potential to reduce nitrates, may suggest a role in C-N cycling (88). All three families transport various amino acids as well as phospholipids and heme. The exchange of amino acids between symbiont and sponge host has previously been observed (89) and may provide the *Tethybacterales* with a competitive advantage over other sympatric microorganisms (90) and possibly allow the sponge hosts to regulate the symbioses via regulation of the quantity of amino acids available for symbiont uptake (91). Similarly, the transfer of heme in the iron-starved ocean environment between sponge host and symbiont could provide a selective advantage, as heme may act as a supply of iron (92). The *Tethybacteraceae* were distinct from the other two families in their potential to transport sugars. As mentioned earlier, the transport of sugars plays an important role in symbiotic interactions (84, 93–95), and it is possible that this family of symbionts require sugars from their sponge hosts.

10.1128/mBio.01577-21.10 TABLE S7 KEGG annotation counts for all *Tethybacterales* and *Poribacteria* genomes grouped by metabolic pathway. Download Table S7, TXT file, 0.3 MB. Copyright © 2021 Waterworth et al. 2021 Waterworth et al. https://creativecommons.org/licenses/by/4.0/ This content is distributed under the terms of the Creative Commons Attribution 4.0 International license.

### Comparative analyses of functional potential between *Tethybacterales* and *Poribacteria*.

**FIG 3:**
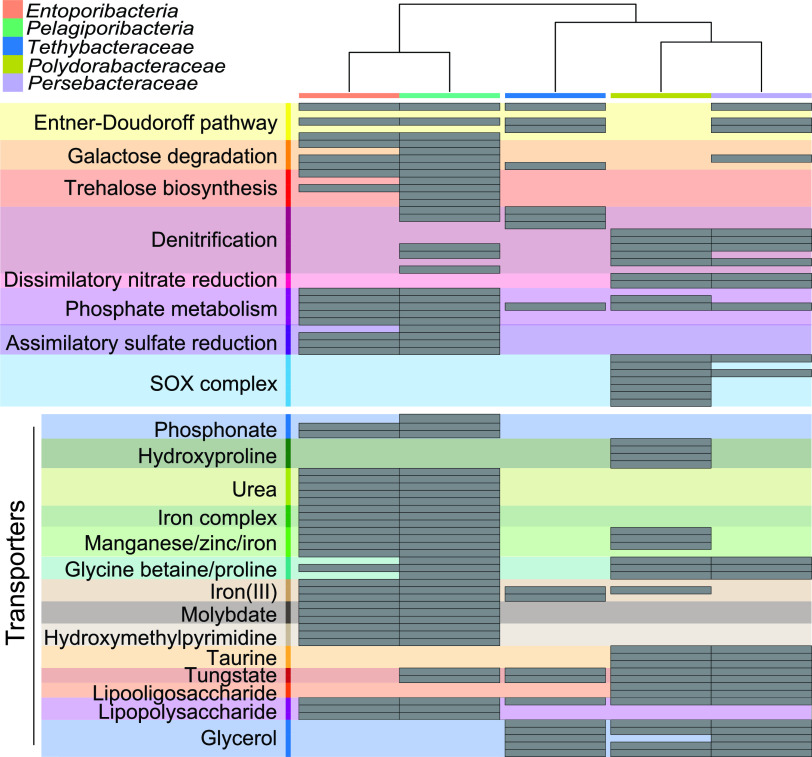
Notable functional differences between *Tethybacterales* and *Poribacteria*. A summary of the most significant differences in the functional gene repertoires of bacterial families with the *Poribacteria* and *Tethybacterales*. The presence of metabolic genes (KEGG annotations) detected in any *Tethybacterales* and *Poribacteria* genome bins is indicated with a gray block.

We wanted to determine whether broad-host range sponge-associated symbionts have converged to perform similar roles in their sponge hosts. Accordingly, we annotated 62 *Poribacteria* genomes, which consisted of 24 *Pelagiporibacteria* (free-living) and 38 *Entoporibacteria* (sponge-associated) genomes, and the 17 *Tethybacterales* genomes against the KEGG database. We catalogued the presence/absence of 896 unique genes spanning carbohydrate metabolism, methane metabolism, nitrogen metabolism, sulfur metabolism, phosphate metabolism, and several transporter systems (Table S7). Inspection of the functional potential in the *Tethybacterales* and *Poribacteria* revealed several insights (Fig. 3). The gene repertoires of the *Poribacteria* and the *Tethybacterales* are distinct from one another (Fig. S3; Table 2), with notable differences, including the genes associated with dissimilatory nitrate reduction, thiosulfate oxidation, and transport of glycine betaine/proline, glycerol, taurine, tungstate, and lipooligosaccharides, all of which are present in at least two of the three *Tethybacterales* families and absent in the *Poribacteria* (Fig. 3). Conversely, several gene clusters were detected in the *Poribacteria* and absent in the *Tethybacterales*, including trehalose biosynthesis, galactose degradation, phosphate metabolism, assimilatory sulfate reduction, and transport of phosphonate, urea, iron complexes, molybdate, and hydroxymethylpyrimidine (Fig. 3). It has been reported that both *Entoporibacteria* and *Pelagiporibacteria* include genes associated with denitrification (36); however, we could not detect many genes associated with nitrogen metabolism in our analyses (Fig. 3).

**TABLE 2:** Pairwise ANOSIM of presence/absence of KEGG-annotated functional genes in *Poribacteria* and *Tethybacterales*

Taxonomy A	Taxonomy B	*P* value	*R* statistic
*Entoporibacteria*	*Kalypsobacteraceae*	0.0001	0.9819
*Entoporibacteria*	*Pelagiporibacteria*	0.0001	0.5248
*Entoporibacteria*	*Persebacteraceae*	0.0001	0.9929
*Entoporibacteria*	*Tethybacteraceae*	0.0001	0.9581
*Kalypsobacteraceae*	*Pelagiporibacteria*	0.0007	0.4909
*Kalypsobacteraceae*	*Persebacteraceae*	0.0015	0.4776
*Kalypsobacteraceae*	*Tethybacteraceae*	0.0033	0.8915
*Pelagiporibacteria*	*Persebacteraceae*	0.0033	0.5301
*Pelagiporibacteria*	*Tethybacteraceae*	0.0663	0.3124
*Persebacteraceae*	*Tethybacteraceae*	0.0082	0.5938

10.1128/mBio.01577-21.3 FIG S3 The potential functional ability of the three *Tethybacterales* families and the two lineages within the *Poribacteria* appears to be distinct. A nonmetric multidimensional scaling (NMDS) plot of the presence/absence metabolic counts from the *Tethybacterales* and *Poribacteria* calculated using Bray-Curtis distance. Download FIG S3, EPS file, 1.3 MB. Copyright © 2021 Waterworth et al. 2021 Waterworth et al. https://creativecommons.org/licenses/by/4.0/ This content is distributed under the terms of the Creative Commons Attribution 4.0 International license.

We cross-checked gene annotations generated using Prokka (HAMAP database) and BLAST (nonredundant [nr] database). Genes associated with assimilatory nitrate reduction (*narB* and *nirA*) were identified in *Poribacteria* using these alternate annotations, but we could not detect genes associated with denitrification in the *Poribacteria*. Conversely, genes associated with denitrification (*napAB* and *nirK*) were detected in the *Persebacteraceae* of the *Tethybacterales* in Prokka, BLAST, and KEGG annotations (Fig. 3), indicating that their absence in *Poribacteria* genomes was not an artifact of our analyses.

Pairwise analysis of similarity (ANOSIM) (using Bray-Curtis distance) confirmed that the functional genetic repertoire (KEGG annotations) of the *Tethybacterales* bacteria showed a strong, significant dissimilarity to that of the sponge-associated *Entoporibacteria* and the free-living *Pelagiporibacteria* (Table 2). In addition, the *Polydorabacteraceae* and the *Persebacteraceae* were significantly different from one another, but the lower R statistic would suggest that the dissimilarity is not as strong as that between other groups in this analysis, while the *Tethybacteraceae* appear to be more functionally distinct from the other two *Tethybacterales* families.

Taken together, these data suggest that the three *Tethybacterales* families and the *Entoporibacteria* lineages may each fulfil distinct functional or ecological niches within a given sponge host. We then considered the sponge hosts themselves and found that *Entoporibacteria* included in this study associate exclusively with high-microbial abundance (HMA) sponges, while the *Tethybacterales* largely associate with low-microbial abundance (LMA) sponges (Table 3). This difference is consistent with previous findings that LMA and HMA sponges have different bacterial community structures, where the HMA sponges are associated with highly abundant, highly diverse, and similar bacterial communities across all sponges, and LMA sponges have fewer bacterial cells, with lower diversity and often a dominant single population (96–98). More specifically, *Poribacteria* have been identified as “indicator species” for HMA sponges, and *Betaproteobacteria* (now within the *Gammaproteobacteria* class) were indicator species of LMA sponges (97, 99). However, exceptions to *Tethybacterales* associating exclusively with LMA sponges were observed. First, the Iophon methanophila sponges which harbor symbionts within the *Tethybacteraceae* family do not conform to the LMA/HMA dichotomy (100), and second, some sponges, such as *C. singaporensis* (HMA), can play host to both *Tethybacterales* and *Entoporibacteria* species (Table 3), which provides further evidence that these symbionts may serve different purposes within their sponge host. However, why and how these different sponge types select for different broad-host range symbionts remain to be discovered.

**TABLE 3:** *Tethybacterales* and *Entoporibacteria* appear to selectively associate with LMA and HMA sponges, respectively^*a*^

Symbiont order	Symbiont family	Sponge host	Host order	LMA/HMA status	Collection site
*Tethybacterales*	*Persebacteraceae*	*Tsitsikamma* (*T.*) *favus*	*Poecilosclerida*	LMA	Algoa Bay, South Africa
*Tethybacterales*	*Persebacteraceae*	*Crella incrustans*	*Poecilosclerida*	LMA (96, 97)	Bare Island, NSW, Australia
*Tethybacterales*	*Persebacteraceae*	*Crambe crambe*	*Poecilosclerida*	LMA (101)	Cala Montgo, Spain
*Tethybacterales*	*Persebacteraceae*	*Scopalina* sp.	*Scopalinida*	LMA (96, 97)	Bare Island, NSW, Australia
*Tethybacterales*	*Persebacteraceae*	*Tethya stolonifera*	*Tethyida*	LMA (95)	Jones Bay, Tawharanui Peninsula, New Zealand
*Tethybacterales*	*Polydorabacteraceae*	*Coelocarteria singaporensis*	*Poecilosclerida*	HMA (96)	Dobu “bubble” site, Papua New Guinea
*Tethybacterales*	*Polydorabacteraceae*	*Cinachyrella sp.*	*Tetractinellida*	Either is possible for an unknown species (96, 97, 101)	Penghu, Taiwan
*Tethybacterales*	*Tethybacteraceae*	*Iophon methanophila*	*Poecilosclerida*	Hybrid (99)	Chapopote Knoll, Gulf of Mexico
*Tethybacterales*	*Tethybacteraceae*	*Cymbastela concentrica*	*Axinellida*	LMA (100)	Bare Island, Botany Bay, Australia
*Tethybacterales*	*Tethybacteraceae*	*Amphimedon queenslandica*	*Haplosclerida*	LMA (100)	Shark Bay, Heron Island, Great Barrier Reef, Australia
*Entoporibacteria*	NA	Agelas tubulata	*Agelasida*	HMA (95–97, 101)	St. Thomas, Virgin Islands
*Entoporibacteria*	NA	*Melophlu*s sp.	*Tetractinellida*	HMA (102)	Western Shoals, Apra Harbor, Guam
*Entoporibacteria*	NA	*Pseudoceratina* sp.	*Verongiida*	HMA (96)	San Louis Beach, Santa Rita, Guam
*Entoporibacteria*	NA	*Porites lutea* coral	*Scleractinia*	NA	Orpheus Island, Australia
*Entoporibacteria*	NA	*Coelocarteria singaporensis*	*Poecilosclerida*	HMA	D’Entrecasteaux Islands, Papua New Guinea
*Entoporibacteria*	NA	Ircinia ramosa	*Dictyoceratida*	HMA (95–97, 101)	Great Barrier Reef, Australia

a NA, taxonomy not defined at family level.

**FIG 4:**
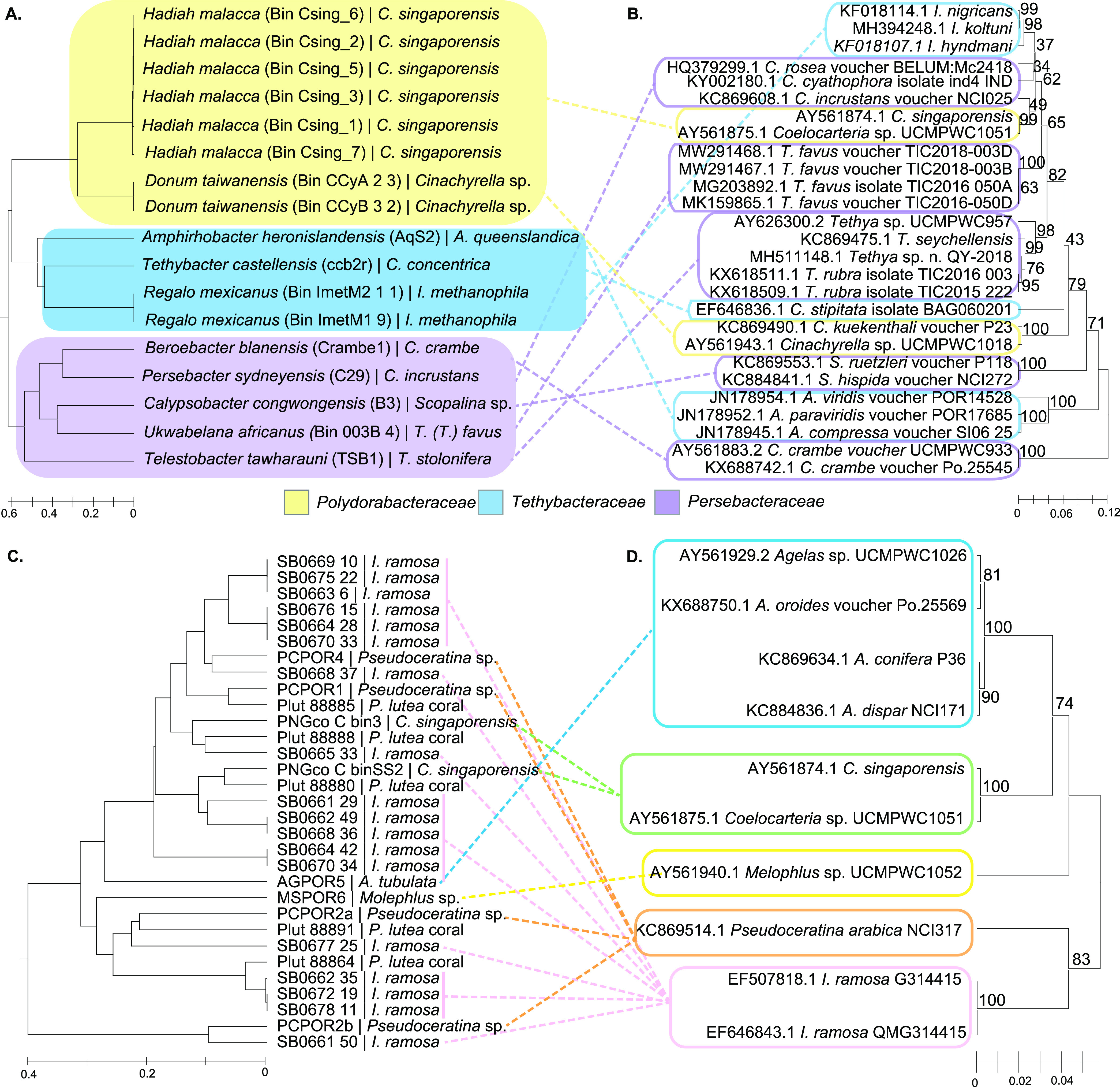
The divergence pattern of sponge-associated *Tethybacterales*, *Entoporibacteria*, and their respective host sponges. The divergence of the *Tethybacterales* and *Entoporibacteria* is incongruent with the phylogeny of the host sponges. (A and C) The branch length of symbiont divergence estimates is proportional to the pairwise rate of synonymous substitution calculated (ML estimation) using a concatenation of genes common to all genomes. The rate of synonymous substitution was calculated using PAL2NAL and CodeML from the PAML package and visualized in MEGA X. (B and D) The phylogeny of host sponges (or close relatives thereof) was inferred with 28S rRNA sequence data using the UPGMA method and maximum composite likelihood model with 1,000 bootstrap replicates. Branch lengths indicate the number of substitutions per site. All ambiguous positions were removed for each sequence pair (pairwise deletion option). Evolutionary analyses were conducted in MEGA X.

We investigated the respective approximate divergence pattern of the *Tethybacterales* and the *Entoporibacteria* and whether their divergence followed that of their sponge hosts. The 18 homologous genes shared between the *Tethybacterales* were used to estimate the rate of synonymous substitution, which provides an approximation for the pattern of divergence between the species (101). We found that the estimated divergence pattern of the *Tethybacterales* (Fig. 4A) and the phylogeny of the host sponges (Fig. 4B) was incongruent. Phylogenetic trees inferred using single-copy marker genes (Fig. 1) and the comprehensive 16S rRNA tree published by Taylor and colleagues (45) confirm this lack of congruency between symbiont and host phylogeny. Other factors, such as collection site or depth, could not explain the observed trend. Similar incongruence of symbiont and host phylogeny was observed for the *Entoporibacteria* (34 homologous genes used to estimate synonymous substitution rates) (Fig. 4C and D), in agreement with previous phylogenetic studies (34, 36, 37). This would suggest that these sponges likely acquired a free-living *Tethybacterales* common ancestor at different time points throughout their evolution and that the same is true for the *Entoporibacteria*. Evidence of coevolution of betaproteobacteria symbionts within sponge families (49, 55, 56, 102) implies that *Tethybacterales* symbionts were likely acquired horizontally at various time points and may have coevolved with their respective hosts subsequent to acquisition.

Finally, the estimated rates of synonymous substitution of homologous genes were used to estimate the relative times at which the *Tethybacterales* and *Entoporibacteria* taxa began diverging. Regardless of the substitution rate used, it was found that the sponge-associated *Tethybacterales* genomes began diverging from one another before the *Entoporibacteria* began diverging from one another (Table S4). If one accepts that divergence between exclusively sponge-associated bacterial lineages began when the common ancestor first associated with a sponge host, then the earlier divergence of sponge-associated *Tethybacterales* (relative to the *Entoporibacteria*) suggests that the *Tethybacterales* may have associated with sponges before the *Poribacteria* common ancestor and represent a more ancient symbiont. However, this hypothesis may prove false if additional *Entoporibacteria* lineages are discovered and added to the analyses, or other factors such as mutation rates, time between symbiont acquisition, and transition to vertical inheritance of symbionts or fossil records disprove this hypothesis.

10.1128/mBio.01577-21.7 TABLE S4 Pairwise synonymous substitution rates (dS) between genomes within the sponge-associated *Tethybacterales* and *Entoporibacteria*. Download Table S4, TXT file, 0.02 MB. Copyright © 2021 Waterworth et al. 2021 Waterworth et al. https://creativecommons.org/licenses/by/4.0/ This content is distributed under the terms of the Creative Commons Attribution 4.0 International license.

### Conclusion.

Here, we have shown that the family to which a broad-host range symbiont belongs dictates the functional potential of the symbiont. This work has expanded our understanding of the *Tethybacterales* and the possible functional specialization of the families within this new order. The *Tethybacterales* are functionally distinct from the *Poribacteria*, which would suggest that although these bacteria are both ubiquitously associated with a wide range of sponge hosts, they likely have not converged to fulfil the same role. Instead, it would appear that these symbionts were selected by the various sponge hosts for existing functional capabilities that fulfil requirements of either HMA or LMA sponges. The phylogenetic incongruence of both *Tethybacterales* and *Entoporibacteria* and their respective sponge hosts suggests that their ancestors were horizontally acquired at different evolutionary time points, and coevolution may have occurred following the establishment of the association. Estimates of when the *Tethybacterales* and *Entoporibacteria* began diverging from their respective common ancestors implied that *Tethybacterales* may have associated with a sponge host before the *Entoporibacteria*, and therefore the *Tethybacterales* may be an older sponge-associated symbiont. However, additional data are required to validate or disprove this hypothesis.

## MATERIALS AND METHODS

### Sponge collection and taxonomic identification.

Sponge specimens *Tsitsikamma* (*Tsitsikamma*) *favus* TIC2016-050A and TIC2016-050C were collected in June 2016 at Evans Peak (33.84548° S, 25.31663° E) at a depth of 20 m via self-contained underwater breathing apparatus (SCUBA). Sponge specimens *T.* (*T.*) *favus* TIC2018-003B and TIC2018-003D were collected in March 2018 at Evans Peak (33.84213° S, 25.81655° E) at a depth of 25 m via SCUBA. Collection permits were acquired prior to collection from the Department Environmental Affairs (DEA) and the Department of Environment, Forestry, and Fisheries (DEFF) under the following permit numbers: in 2015, RES2015/16 and RES2015/21; in 2016, RES2016/11; in 2017, RES2017/43; and in 2018, RES2018/44. Sponge specimens were stored on ice during collection and, thereafter, at −20°C. Subsamples collected for DNA extraction were preserved in RNALater (Invitrogen) and stored at −20°C. Sponge specimens were dissected, thin sections were generated, and spicules were mounted on microscope slides and examined to allow species identification, as done previously (103–105). Molecular barcoding (28S rRNA gene) was also performed for several of the sponge specimens (Fig. S1) as described previously (56).

10.1128/mBio.01577-21.1 FIG S1 Phylogeny of sponges within the family *Latrunculiidae*. Sponge phylogeny was inferred using 28S rRNA sequence data using the maximum-likelihood method and the Tamura-Nei model with 1,000 bootstrap replicates. Branch lengths indicate the number of substitutions per site. All ambiguous positions were removed for each sequence pair (pairwise deletion option). Evolutionary analyses were conducted in MEGA X. Download FIG S1, EPS file, 1.1 MB. Copyright © 2021 Waterworth et al. 2021 Waterworth et al. https://creativecommons.org/licenses/by/4.0/ This content is distributed under the terms of the Creative Commons Attribution 4.0 International license.

### Metagenomic sequencing and analysis.

Small sections of each preserved sponge (approximately 2 cm^3^) were pulverized in 2 ml sterile artificial seawater (24.6 g NaCl, 0.67 g KCl, 1.36 g CaCl_2_·2H_2_O, 6.29 g MgSO_4_·7H_2_O, 4.66 g MgCl_2_·6H_2_O, 0.18 g NaHCO_3_, and distilled H_2_O to 1 liter) with a sterile mortar and pestle. The resultant homogenate was centrifuged at 16,000 rpm for 1 min to pellet cellular material. Genomic DNA (gDNA) was extracted using the ZR fungal/bacterial DNA miniprep kit (D6005; Zymo Research). Shotgun metagenomic sequencing was performed for four *T.* (*T.*) *favus* sponge specimens using Ion Torrent platforms. Shotgun metagenomic libraries, of reads 200 bp in length, were prepared for each of the four sponge samples (TIC2016-050A, TIC2018-003B, TIC2016-050C, and TIC2018-003D) using an Ion P1.1.17 chip. Additional sequence data of 400 bp were generated for TIC2016-050A using an Ion S5 530 chip. TIC2016-050A served as a pilot experiment, and we wanted to identify which read length was best for our investigations. However, we did not want to waste additional sequence data and included it when assembling the TIC2016-050A metagenomic contigs, so the 400-bp reads were included in the assembly of these metagenomes. Metagenomic data sets were assembled into contiguous sequences (contigs) with SPAdes v3.12.0 (106) using the –iontorrent and –only-assembler options. Contigs that were classified as bacterial were selected and clustered into genomic bins using Autometa (107) and manually curated for optimal completion and purity. Validation of the bins was performed using CheckM v1.0.12 (108). Of the 50 recovered genome bins, 5 were of high quality, 13 were of medium quality, and 32 were of low quality in accordance with MIMAG standards (62) (Table S1).

### Acquisition and assembly of reference genomes.

The genome of *A. queenslandica* symbiont Aqs2 (GCA_001750625.1) was retrieved from the NCBI database. Similarly, other sponge-associated *Tethybacterales* MAGs from the JGI database were downloaded and used as references (3300007741_3, 3300007056_3, 3300007046_3, 3300007053_5, 3300021544_3, 3300021545_3, 3300021549_5, 2784132075, 2784132054, 2814122900, 2784132034, and 2784132053).

A total of 36 raw-read SRA data sets from sponge metagenomes were downloaded from the SRA database (Table S2). Illumina reads from these data sets were trimmed using Trimmomatic v0.39 (109) and assembled using SPAdes v3.14 (106) in –meta mode. Contigs classified as bacterial were selected and used for further binning using Autometa (107). This resulted in a total of 393 additional genome bins (Table S1), the quality of which was assessed using CheckM (108) and taxonomically classified with GTDB-Tk (110) with database release 95. A total of 27 bins were classified as AqS2 and were considered likely members of the newly proposed *Tethybacterales* order (45). However, 10 of the 27 bins were low quality and were not used in downstream analyses. In addition, 59 *Poribacteria* genome bins were downloaded from the NCBI database for functional comparison (Table S3), and three were used from the 393 genome bins generated in this study (Geodia parva sponge hosts).

10.1128/mBio.01577-21.5 TABLE S2 SRR accession numbers for all Illumina metagenomic data sets assembled and binned in this study. Download Table S2, TXT file, 0.002 MB. Copyright © 2021 Waterworth et al. 2021 Waterworth et al. https://creativecommons.org/licenses/by/4.0/ This content is distributed under the terms of the Creative Commons Attribution 4.0 International license.

10.1128/mBio.01577-21.6 TABLE S3 Accession numbers and metadata for all *Poribacteria* genomes analyzed in this study. Download Table S3, TXT file, 0.02 MB. Copyright © 2021 Waterworth et al. 2021 Waterworth et al. https://creativecommons.org/licenses/by/4.0/ This content is distributed under the terms of the Creative Commons Attribution 4.0 International license.

### Taxonomic identification.

Partial and full-length 16S rRNA gene sequences were extracted from bins using barrnap 0.9 (https://github.com/tseemann/barrnap). Extracted sequences were aligned against the nr database using BLASTn (111). Genomes were additionally uploaded individually to autoMLST (112) and analyzed in both placement mode and *de novo* mode (IQ tree and ModelFinder options enabled and concatenated gene tree selected). All bins and downloaded genomes were taxonomically identified using GTDB-Tk (110).

### Genome annotation and metabolic potential analysis.

All bins and downloaded genomes were annotated using Prokka v1.13 (113) with NCBI compliance enabled. Protein-coding amino-acid sequences from genomic bins were annotated against the KEGG database using kofamscan (114) with output in mapper format. Custom Python scripts were used to summarize annotation counts (find scripts here: https://github.com/samche42/Family_matters). Potential biosynthetic gene clusters (BGCs) were identified by uploading genome bins to the antiSMASH Web server (115) with all options enabled. Predicted amino acid sequences of genes within each identified gene cluster were aligned against the nr database using BLASTp (111) to identify the closest homologs. Protein sequences of genes within each identified gene cluster were aligned against the nr database using BLASTp (111) to identify the closest homolog.

### Phylogeny and function of *Tethybacterales* species.

A subset of orthologous genes common to all medium-quality *Tethybacterales* genomes/bins was created. Shared amino acid identity (AAI) was calculated with the aai.rb script from the enveomics package (116). 16S rRNA genes were analyzed using BLASTn (111). Functional genes were annotated against the KEGG database using kofamscan (114). Annotations were collected into functional categories and visualized in R (see https://github.com/samche42/Family_matters for all scripts). A Nonmetric multidimensional scaling (NMDS) plot of the presence/absence metabolic counts was constructed using Bray-Curtis distance using the vegan package (117) in R. Analysis of similarity (ANOSIM) analyses were also conducted using the vegan package in R using Bray-Curtis distance and 9,999 permutations.

### Genome divergence estimates.

Divergence estimates were performed as described previously (118). Briefly, homologous genes in *Tethybacterales* genomes were identified using OMA v2.4.2 (119). A subset of homologous genes present in all genomes was created. Homologous genes were aligned using MUSCLE v3.8.155 (120) and clustered into fasta files representing each genome using merge_fastas_for_dNdS.py (see https://github.com/samche42/Family_matters for all scripts). The corresponding nucleotide sequences were extracted from Prokka annotations using multifasta_seqretriever.py. All stop codons were removed using remove_stop_codons.py. All nucleotide sequences, per genome, were concatenated to produce a single nucleotide sequence per genome using the union function from EMBOSS (121). All amino acid sequences were similarly concatenated. This resulted in a single concatenated nucleotide sequence and a single concatenated amino acid sequence per genome. Concatenated nucleotide sequences were clustered into two fasta files (one nucleotide, one protein sequence) and then aligned using PAL2NAL (122). The resultant alignment was then run in codeml to produce pairwise synonymous substitution rates (dS). Divergence estimates can be determined by dividing pairwise dS values by a given substitution rate (substitutions per year) and be further divided by 1 million to provide estimates of branch divergence million years ago (mya). Pairwise synonymous substitution rates can be found in Table S4. Pairwise divergence values were illustrated as a tree using MEGA X (123). Concatenated amino acid and nucleotide sequences of the 18 orthologous genes were aligned using MUSCLE v3.8.155 (120), and the evolutionary history was inferred using the UPMGA method (124) in MEGA X (123) with 10,000 bootstrap replicates.

### Identification of unique and host-associated genes in putative symbiont genome bins.

A custom database of genes from all bacterial bins (with the exception of the putative *Tethybacterales* symbionts) was created using the “makedb” option in DIAMOND (125) to identify genes that were unique to the putative *Tethybacterales* symbionts. To be exhaustive and screen against the entire metagenome, genes from low-quality genomes (except low-quality putative *Tethybacterales* genomes), small contigs (<3000 bp) that were not included in binning, and unclustered contigs (i.e., included in binning but not placed within a bin) were included in this database. Putative *Tethybacterales* genes were aligned using DIAMOND blast (125). A gene was considered “unique” if the aligned hit shared less than 40% amino acid identity with any other genes from the *T.* (*T.*) *favus* metagenomes and had no significant hits against the nr database or were identified as pseudogenes. All “unique” putative *Tethybacterales* genes annotated as “hypothetical” (both Prokka and NCBI nr database annotations) were removed. Finally, we compared Prokka annotation strings between the putative *Tethybacterales* bins and all other *T.* (*T.*) *favus*-associated genome bins and excluded any putative *Tethybacterales* genes that were found to have the same annotation as a gene in one of the other bins.

### Data availability.

The raw 16S amplicon and metagenomic read data can be accessed from the NCBI website under BioProject PRJNA508092.
